# P-1736. Temporal Shifts in Candidemia Epidemiology in Costa Rica: Pre-Pandemic, Pandemic, and Post-Pandemic Analysis (2007-2023)

**DOI:** 10.1093/ofid/ofaf695.1907

**Published:** 2026-01-11

**Authors:** Jose A Castro Cordero, Juan Villalobos Vindas, Elvira Segura Retana, Heylin Estrada Murillo, Alvaro A Aviles Montoya, Carlos Ramírez Valverde, Saúl Quirós Cárdenas, Laura Villalobos González

**Affiliations:** Caja Costarricense de Seguro Social, Uruca, San Jose, Costa Rica; Caja Costarricense de Seguro Social, Uruca, San Jose, Costa Rica; Caja Costarricense de Seguro Social, Uruca, San Jose, Costa Rica; Caja Costarricense del Seguro Social, La Unión, Cartago, Costa Rica; Caja Costarricense de Seguro Social, Uruca, San Jose, Costa Rica; Caja Costarricense del Seguro Social, La Unión, Cartago, Costa Rica; CCSS, San Jose, San Jose, Costa Rica; Caja Costarricense de Seguro Social, Uruca, San Jose, Costa Rica

## Abstract

**Background:**

The epidemiology of candidemia has shown significant variations over time. This study examines the impact of the COVID-19 pandemic on candidemia patterns in Costa Rica.

**Figure:**
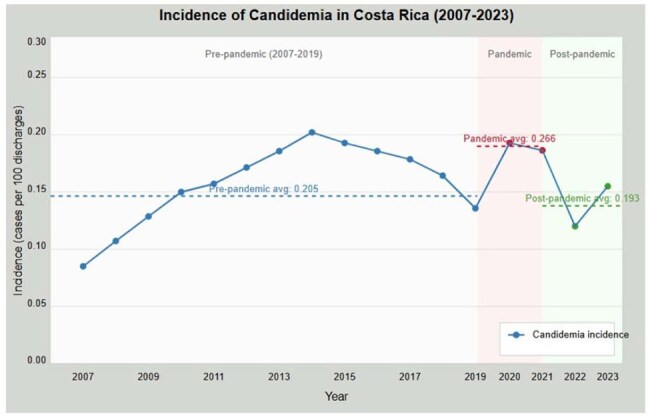
Incidence of Candidemia Incidence of Candidemia in Costa Rica (2007-2023)

**Figure:**
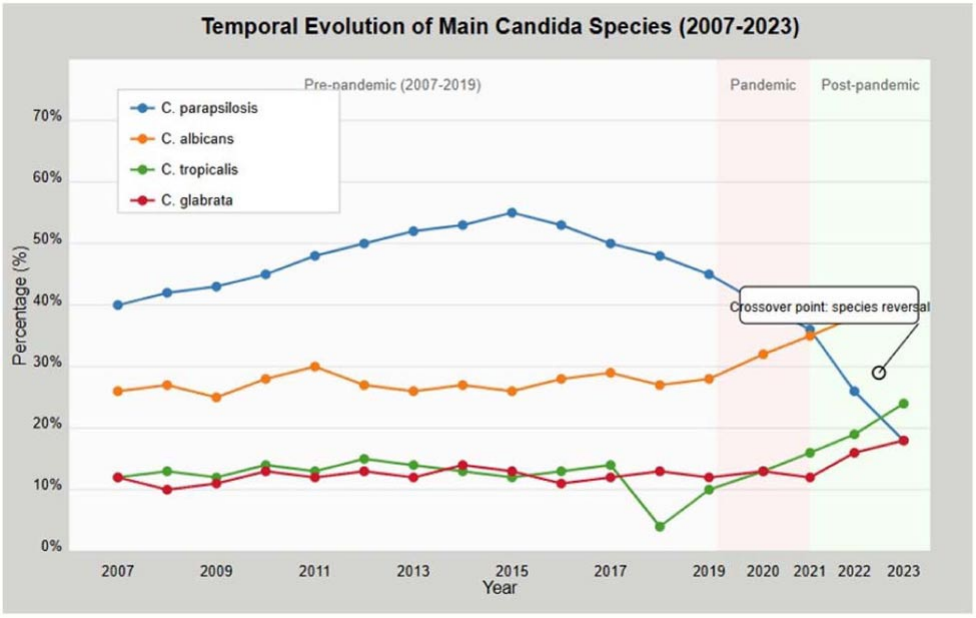
Temporal Evolution Temporal Evolution of Main Candida Species (2007-2023)

**Methods:**

We analyzed 2,128 candidemia cases from two tertiary hospitals in Costa Rica across three distinct periods: pre-pandemic (2007-2019, n=1,658), pandemic (2020-2021, n=256), and post-pandemic (2022-2023, n=214).

**Results:**

Candidemia incidence showed significant temporal variations: increasing from 0.119/100 discharges (2007) to 0.283/100 (2014), followed by fluctuations until rising significantly during the pandemic (0.266/100 discharges vs. 0.205/100 pre-pandemic), and subsequently decreasing post-pandemic (0.193/100). The pandemic marked a critical inflection point in species distribution (χ²=73.41, p< 0.001). C. parapsilosis dominance (47.8% pre-pandemic) diminished progressively (40.6% during pandemic, 29.0% post-pandemic), while C. albicans increased (29.7%, 35.2%, and 40.2%, respectively). C. tropicalis showed sustained increases from pre-pandemic (7.8%) to post-pandemic periods (14.5%). Mixed candidemia cases increased from none (2007-2010) to 5.6% (2023), with notable absence during 2021.

**Conclusion:**

The COVID-19 pandemic represents a significant inflection point in candidemia epidemiology in Costa Rica, with persistent post-pandemic shifts in species distribution. The emergence of C. albicans as the dominant species over C. parapsilosis post-pandemic represents a significant epidemiological transition with important clinical implications for empirical treatment strategies and infection control practices.

**Disclosures:**

All Authors: No reported disclosures

